# Epigenetic modifications of interleukin-6 in synovial fibroblasts from osteoarthritis patients

**DOI:** 10.1038/srep43592

**Published:** 2017-03-06

**Authors:** Fei Yang, Song Zhou, Chuandong Wang, Yan Huang, Huiwu Li, You Wang, Zhenan Zhu, Jian Tang, Mengning Yan

**Affiliations:** 1 Shanghai Key Laboratory of Orthopaedic Implants, Department of Orthopaedics, Shanghai Ninth People’s Hospital, Shanghai Jiao Tong University School of Medicine, 639 Zhizaoju Road, Shanghai 200011, P. R. China; 2 Department of Orthopaedics, Linyi People’s Hospital, 27 East Jiefang Road, Linyi 276000, P. R. China; 3 The Key Laboratory of Stem Cell Biology, Institute of Health Sciences, Shanghai Institutes for Biological Sciences, Chinese Academy of Sciences & Shanghai Jiao Tong University School of Medicine, 320 Yueyang Road, Shanghai 200031, P. R. China

## Abstract

Osteoarthritis (OA) is the most common degenerative disease of the synovial joint. The synovial membrane is responsible for the inflammatory reaction leading to the secretion of macrophage-derived pro-inflammatory cytokines, such as IL-6. Suppressing IL-6 over-expression in synovial fibroblasts (SF) is a promising method to prevent OA development and progression, in which the prerequisite is the elucidation of the molecular mechanisms underlying IL-6 over-expression in SF. Currently, there are few reports concerning epigenetic modifications in IL-6 in OA SF. In the present study, we attempted to investigate this phenomenon. SF over-expressing IL-6 was collected from OA patients. DNA hypomethylation and histone hyperacetylation were observed in the IL-6 promoter regions in OA SF compared with normal SF. No differences in the status of H3K9 di-methylation, H3K27 tri-methylation and H3K4 tri-methylation were observed in the IL-6 promoter regions between normal and OA SF. DNA (cytosine-5-)-methyltransferase 3 alpha (Dnmt3a) overexpression and anacardic acid (histone acetyltransferase inhibitor) treatment increased DNA methylation and decreased histone acetylation in the IL-6 promoter, and IL-6 over-expression in OA SF was suppressed. These observations provide deeper insight into the pathogenesis of OA and can be used to design new drugs and develop new therapeutic methods to treat OA.

Osteoarthritis (OA) is the most prevalent degenerative disease affecting synovial joint. Its pathological phenotypes include articular cartilage impairment, subchondral bone sclerosis, bone resorption/formation imbalance, osteophyte formation, and inflammation of the synovial membrane^1^,^2^. Its symptoms include pain, stiffness and swelling of involved joints, and muscle weakness. Although the precise etiology of OA remains unknown^3^, the consensus has been built that the synovial membrane plays an important role in the joint chronic inflammation, neovascularization and cartilage destruction in which macrophage-derived pro-inflammatory cytokines, such as IL-1β, IL-6, IL-8 and TNF-α, are involved^4^,^5^,^6^.

Inflammation is one of the most important factors in OA pathophysiology^7^, in which elevated production of cytokines, such as IL-6, is always characterized. IL-6 with numerous biological functions is regarded as the major player that regulates the innate immune response, hemopoiesis, and inflammation. In skeleton system, IL-6 activates osteoclasts and stimulates the synovium to produce matrix metalloproteinases (MMPs) that are responsible for degrading cartilage in OA^8^. Blocking IL-6 over-expression in synovial fibroblasts (SF) is believed to be a promising method to prevent OA progression, in which the prerequisite is the elucidation of molecular mechanisms underlying IL-6 over-expression in SF.

Mammalian gene expression is tightly modulated by distinct epigenetic modifications including DNA methylation, histone modifications and miRNAs^9^. DNA methylation, generally leading to transcription silence, is one of the best studied epigenetic modifications, particularly in oncogenesis. In addition to DNA methylation, histone modifications also play an essential role in gene transcription. They include methylation, acetylation, phosphorylation, SUMOylation and ubiquitination so on. These histone modifications regulate gene transcription through either directly modulating chromatin structure or indirectly providing platforms for transcriptional factors. Among the various forms of histone modifications, acetylation is generally associated with gene transcription activation, whereas the role of methylation depends specifically on the site and number of methyl groups (i.e., mono-, di- or tri-methylation). For example, mono-methylation of histone H3 lysine (K) 4 is observed in the enhancer elements in the human genome, and tri-methylation mainly marks active promoters^10^. In contrast, H3 K9 and K27 tri-methylation have been regarded as repressive markers^10^.

There is a body of literature with respect to epigenetic regulation of genes in OA. These studies mainly focus on cartilage. For example, the promoters of genes encoding catabolic factors such as MMP3, 9, 13, and ADAMTS4 are demethylated, which contributed to the upregulation of these genes in OA-related pathogenic conditions^11^. The SIR2 family, one of two groups of histone deacetylases (HDACs), includes seven family members in mammals (SIRT1-7)^12^. In chondrocytes, SIRT1 was observed to activate the expression of cartilage-specific genes such as Col2a1 by forming a complex with Sox9 and targeting it for deacetylation^13^. Sox9 is itself subjected to intensive epigenetic regulation during OA progression. Under OA pathogenic conditions, the promoter of Sox9 was observed to have elevated levels of trimethylation of H3K9 and H3K27, and decreased acetylation of H3K9, 15, 18, 23, and 27, which collectively leads to transcriptional repression of Sox9^14^.

Currently, there are few reports concerning epigenetic modifications in OA SF, especially in IL-6 gene. We have hypothesized that the epigenetic modifications in IL-6 promoter in SF were involved in the pathophysilogy of OA. Therefore, in the present study, we attempted to investigate it. SF over-expressing IL-6 was isolated from OA patients. Epigenetic modifications in the IL-6 promoter region, including DNA methylation, histone acetylation and histone methylation, were investigated. In addition, the binding of methyl-CpG binding protein 2 (MeCP2), DNA (cytosine-5-)-methyltransferase 1 (Dnmt1), Dnmt3a, HDAC1, histone acetyltransferase 1 (HAT1), CREB binding protein (CBP) and p300 on the IL-6 promoter regions was also investigated. Further, by altering the status of DNA methylation and histone acetylation in the IL-6 promoter, IL-6 overexpression was suppressed in OA SF.

## Results

### IL-6 over-expression in SF and synovial fluid from OA patients

**Figure 1:**
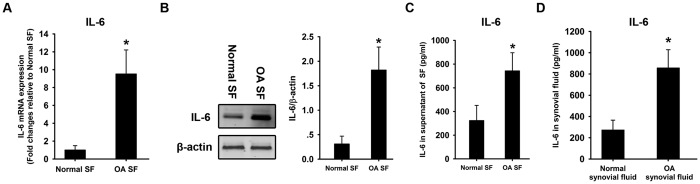
IL-6 over-expression in synovial fibroblasts and synovial fluid from OA patients. Synovial fibroblasts (SF) were isolated from non-arthritic donors and OA patients and cultured *in vitro*. Total RNA was isolated for quantitative RT-PCR using IL-6 primers **(A)** β-actin was used as an internal control. The results are expressed as the fold-change relative to normal SF. Total protein was harvested for western blot analysis using an IL-6 antibody **(B)** β-actin was used as an internal control. IL-6 and β-actin bands density was measured using image software. The relative protein expression level of IL-6 was normalized to β-actin. The supernatants of normal and OA SF were collected and ELISA was employed to measure IL-6 concentration **(C)** The IL-6 level was measured in synovial fluid from non-arthritic donors and OA patients using ELISA **(D)** Data are shown as the means ± SD. *p < 0.05, OA SF *vs.* normal SF, OA synovial fluid *vs.* normal synovial fluid. All of the data were obtained from 20 OA patients receiving knee-replacement surgery and 15 samples of non-arthritic synovial tissues obtained during arthroscopy.

SF was harvested from non-arthritic donors and OA patients and cultured *in vitro*. Total RNA was isolated for quantitative RT-PCR using IL-6 primers. The results revealed the significant upregulation of IL-6 mRNA in OA SF compared with normal controls (Fig. 1A). Total protein was also isolated for western blot analysis using IL-6 antibody. Consistent with the results of RT-PCR, the significant upregulation of IL-6 protein was observed in OA SF compared with normal controls (Fig. 1B). The supernatants of normal and OA SF were collected and ELISA was employed to measure IL-6 concentration. The significantly higher level of IL-6 secretion was observed from OA SF, compared with normal SF (Fig. 1C). Synovial fluid was collected from non-arthritic donors and OA patients. The IL-6 concentration was measured using ELISA. The results revealed higher IL-6 levels in synovial fluid from OA patients compared with normal controls (Fig. 1D).

### DNA hypo-methylation in the IL-6 promoter in SF from OA patients

**Figure 2:**
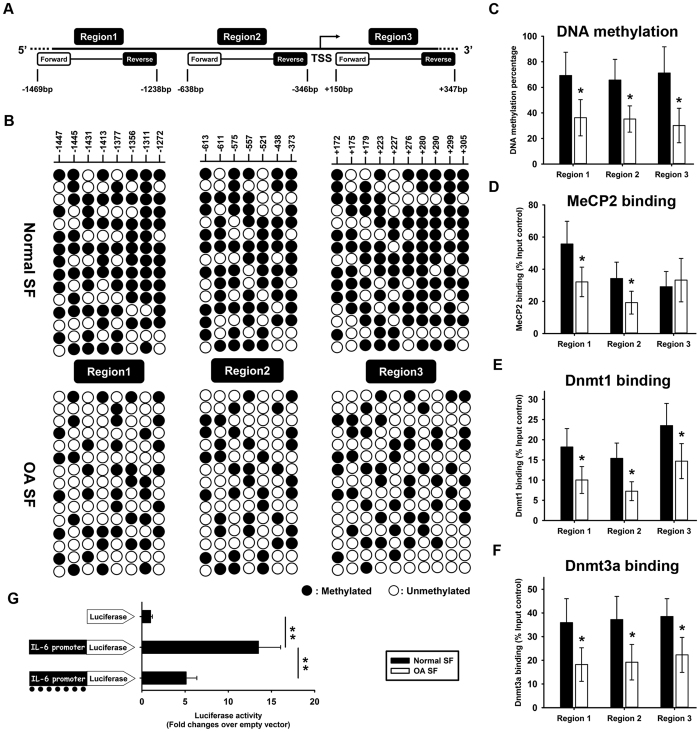
DNA hypo-methylation in the IL-6 promoter in synovial fibroblasts from OA patients. Analysis of DNA methylation in the human IL-6 promoter (−1500 ~+500 bp) revealed three regions containing abundant CpG sites around the transcription start site (TSS) **(A)** Genomic DNA was isolated from normal and OA SF. The DNA methylation statuses of three regions in the IL-6 promoter were investigated **(B)** Open circles indicate unmethylated CpG sites, and closed circles indicate methylated CpG sites. The DNA methylation percentage was calculated, and the results were expressed as the percentage of methylated CpG sites among all of the CpG sites in each region (**C**) ChIP was performed to measure MeCP2 **(D)**, Dnmt1 **(E)** and Dnmt3a **(F)** binding to the IL-6 promoter in normal and OA SF. IgG was used as a negative control. The results are expressed as the percentage of MeCP2 and Dnmt3a binding in the input control. The human IL-6 promoter region (−1500 to +500 bp) was cloned into the pGL3-Basic vector. M*.SssI* was used to methylate CpG sites *in vitro*, and a transient reporter assay was performed in normal SF (**G**) The results are expressed as the fold-change in relative luciferase units (RLUs) relative to the “empty reporter”. Data are shown as the means ± SD. *p < 0.05, **p < 0.01, OA SF *vs.* normal SF. All of the data were obtained from 20 OA patients receiving knee-replacement surgery and 15 samples of non-arthritic synovial tissues obtained during arthroscopy.

MethPrimer computer program (http://www.urogene.org/methprimer/) was employed to analyze the human IL-6 promoter, which contains abundant CpG sites (i.e., potential DNA methylation targets) around the transcription start site (TSS). As shown in Fig. 2A, three regions were selected: region 1 (−1469 ~ −1238 bp, 8 CpG sites), region 2 (−638 ~ −346 bp, 7 CpG sites) and region 3 (+150 ~+347 bp, 10 CpG sites). The DNA sequence is shown in Fig. S1. The methylation status of these CpG sites was investigated in normal and OA SF. Significant DNA hypo-methylation was observed in regions 1 (69% *vs.* 36%), 2 (65% *vs.* 35%), and 3 (71% *vs.* 30%) in OA SF compared with normal controls (Fig. 2B and C).

We also performed ChIP assays to measure MeCP2 binding. IgG was used as a negative control (Fig. S2). MeCP2 specifically binds to the DNA methylation sites in the IL-6 promoter. The results revealed weaker MeCP2 binding in regions 1 and 2 in the IL-6 promoter in OA SF compared with normal controls (Fig. 2D). No difference in MeCP2 binding was observed in region 3 between normal and OA SF (Fig. 2D). We also measured the binding of Dnmt1 (a maintenance enzyme that prefers hemi-methylated DNA) and Dnmt3a, which is responsible for *de novo* adding a methyl group to cytosine on the IL-6 promoter. The results revealed weaker Dnmt1 (Fig. 2E) and Dnmt3a (Fig. 2F) binding within regions 1, 2, and 3 in the IL-6 promoter in OA SF compared with normal controls, although there was no difference in global mRNA expression levels of Dnmt1 and Dnmt3a between normal and OA SF (Fig. S3).

Further, the human IL-6 promoter (−1500 ~+500 bp) was amplified from genomic DNA using PCR and cloned into the pGL3-basic vector. *M.SssI* was utilized to *in vitro* methylate the IL-6 promoter, and transient reporter assays were subsequently performed in normal SF. As shown in Fig. 2G, *in vitro* IL-6 promoter methylation resulted in a significant reduction in luciferase activity.

### Histone hyper-acetylation in the IL-6 promoter in SF from OA patients

**Figure 3:**
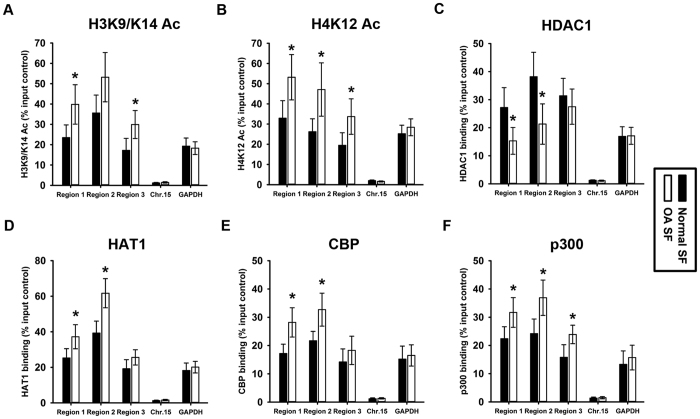
Histone hyper-acetylation in the IL-6 promoter in synovial fibroblasts from OA patients. ChIP assays were used to examine acetylated H3K9/K14 **(A),** H4K12 **(B)**, HDAC1 **(C),** HAT1 **(D),** CBP **(E)** and p300 **(F)** occupancy in three regions of the IL-6 promoter in normal and OA SF. Silent (Chromosome 15) and active (GAPDH) chromatin regions were included as controls in the ChIP-qPCR experiments. The results were normalized to the percentage in the input control. Data are shown as the means ± SD. *p < 0.05, OA SF *vs.* normal SF. All of the data were obtained from 20 OA patients receiving knee-replacement surgery and 15 samples of non-arthritic synovial tissues obtained during arthroscopy.

IL-6 promoter histone modifications were also investigated in normal and OA SF. Histones are important for gene expression. H3 and H4 tails are potentially acetylated at multiple sites, and these acetylation sites are generally associated with gene transcription activation. In the present study, we used ChIP assays to examine the acetylation of these two histone tails in the IL-6 promoter in normal and OA SF; we selected H3K9/K14 and H4K12 acetylation to represent the acetylation of H3 and H4, respectively. Negative control experiments were performed in parallel using IgG. In addition, we included representative silent (Chromosome 15) and active (GAPDH) chromatin regions as controls in the ChIP-qPCR experiments. We observed significant increases in H3K9/K14 (Fig. 3A) and H4K12 (Fig. 3B) acetylation within regions 1, 2, and 3 in the IL-6 promoter in OA SF compared with normal controls. We also found that the GAPDH promoter has more acetylated histone H3 and H4 occupancy than the silent region on Chromosone 15, which correlates positively with gene activity. Further, we performed ChIP assays to measure HDAC1, HAT1, CBP and p300 binding, which is responsible for removing and adding the acetyl groups. The results revealed weaker HDAC1 binding (Fig. 3C) and stronger HAT1 (Fig. 3D) and CBP binding (Fig. 3E) within regions 1 and 2 in the IL-6 promoter in OA SF compared with normal controls. Meanwhile, stronger p300 binding was observed within regions 1, 2 and 3 in the IL-6 promoter in OA SF compared with normal controls (Fig. 3F). It should be noted that there was no difference in global mRNA expression levels of HDAC1 and HAT1 between normal and OA SF (Fig. S4).

### Histone methylation in the IL-6 promoter in SF from OA patients

**Figure 4:**
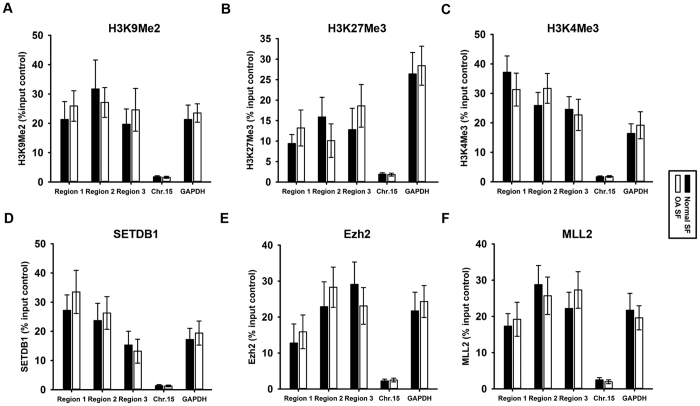
Histone methylation in the IL-6 promoter in synovial fibroblasts from OA patients. ChIP assays were used to examine di-methylated H3K9 **(A),** tri-methylated H3K27 **(B)**, tri-methylated H3K4 **(C)**, SETDB1 **(D)**, Ezh2 **(E)** and MLL2 **(F)** occupancy in three regions of the IL-6 promoter in normal and OA SF. The results were normalized to the percentage in the input control. Data are shown as the means ± SD. All of the data were obtained from 20 OA patients receiving knee-replacement surgery and 15 samples of non-arthritic synovial tissues obtained during arthroscopy.

Depending on the site and degree of histone methylation, it can lead to either gene activation or repression. Di-methylated H3K9 and tri-methylated H3K27 are repressive markers^10^,^15^. Tri-methylated H3K4 is active marker^16^. In this study, we used ChIP assays to examine H3K9 di-methylation, H3K27 tri-methylation and H3K4 tri-methylation in the IL-6 promoter in normal and OA SF. No differences were observed in the distribution of H3K9 di-methylation (Fig. 4A), H3K27 tri-methylation (Fig. 4B) and H3K4 tri-methylation (Fig. 4C) in region 1, 2, and 3 in the IL-6 promoter between normal and OA SF. Further, we measured SETDB1 (responsible for methylating H3K9), Ezh2 (responsible for methylating H3K27) and MLL2 (responsible for methylating H3K4) binding. The results revealed no differences in SETDB1 (Fig. 4D), Ezh2 (Fig. 4E) and MLL2 (Fig. 4F) binding within regions 1, 2, and 3 in the IL-6 promoter between normal and OA SF.

### Suppression of IL-6 over-expression in OA SF through alterations in the status of DNA methylation and histone acetylation in the IL-6 promoter

**Figure 5:**
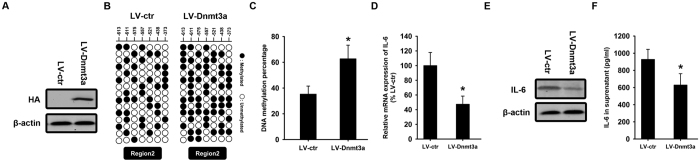
Suppression of IL-6 over-expression in OA synovial fibroblasts after increasing DNA methylation in the IL-6 promoter. OA SF was infected with lentivirus loaded with HA-tagged Dnmt3a **(A)** Empty vector was used as a control. Genomic DNA was isolated from OA SF with Dnmt3a over-expression. The DNA methylation statuses of region 2 in the IL-6 promoter were investigated **(B)** Open circles indicate unmethylated CpG sites and closed circles indicate methylated CpG sites. DNA methylation percentage was calculated, and the results were expressed as the percentage of methylated CpG sites in all of CpG sites in region 2 **(C)** Total RNA was isolated from OA SF with and without Dnmt3a over-expression for quantitative RT-PCR using IL-6 primers **(D)** Total protein was isolated for western blot analysis using an IL-6 antibody **(E)** β-actin was used as an internal control. The supernatants of OA SF with and without Dnmt3a overexpression were collected and ELISA was employed to measure IL-6 concentration **(F)** Data are shown as the means ± SD. *p < 0.05, LV-Dnmt3a *vs.* LV-ctr. All of the data were obtained from 20 OA patients receiving knee-replacement surgery and 15 samples of non-arthritic synovial tissues obtained during arthroscopy.

After observing DNA hypomethylation and histone hyperacetylation in the IL-6 promoter in OA SF, we next determined whether IL-6 over-expression in OA SF could be modulated through alterations of the status of DNA methylation and histone acetylation. To this end, we employed lentivirus loaded with HA-tagged Dnmt3a to infect OA SF (Fig. 5A). In response to Dnmt3a over-expression, the IL-6 promoter was methylated (Fig. 5B and C). Total RNA and protein were harvested from OA SF with and without Dnmt3a over-expression for quantitative RT-PCR and western blot analysis to measure IL-6 expression levels. The results revealed that both the mRNA (Fig. 5D) and protein (Fig. 5E) levels of IL-6 in OA SF could be significantly suppressed after elevating the DNA methylation of the IL-6 promoter. The supernatants of OA SF with and without Dnmt3a overexpression were collected and ELISA was employed to measure IL-6 concentration. The results revealed the significantly lower levels of IL-6 secretion from OA SF with Dnmt3a overexpression, compared to without overexpression (Fig. 5F).

**Figure 6:**
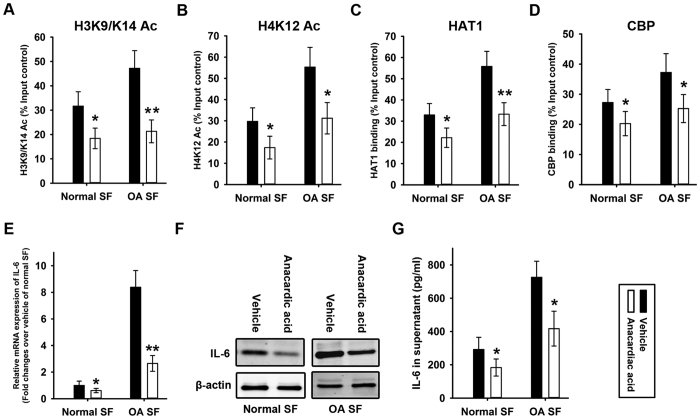
Suppression of IL-6 over-expression in normal and OA synovial fibroblasts by decreasing histone acetylation in the IL-6 promoter. Normal and OA SF was treated with anacardic acid (4 μM). ChIP was performed to measure acetylated H3K9/K14 (**A**), acetylated H4K12 (**B**), HAT1 (**C**) and CBP (**D**) occupancy in region 2 of the IL-6 promoter in normal and OA SF treated with anacardic acid. The results were normalized to the percentage of the input control. Total RNA was isolated from normal and OA SF with and without anacardic acid treatment for quantitative RT-PCR using IL-6 primers (**E**) Total protein was isolated for western blot analysis using an IL-6 antibody (**F**) β-actin was used as an internal control. The supernatants of normal and OA SF with and without anacardic acid treatment were collected and ELISA was employed to measure IL-6 concentration (**G**) Data are shown as the means ± SD. *p < 0.05, **p < 0.01, anacardic *vs.* vehicle. All of the data were obtained from 20 OA patients receiving knee-replacement surgery and 15 samples of non-arthritic synovial tissues obtained during arthroscopy.

In addition, anacardic acid, a histone acetyltransferase inhibitor, was employed to treat normal and OA SF. H3K9/K14 (Fig. 6A) and H4K12 (Fig. 6B) acetylation was inhibited in response to anacardic acid treatment. Accordingly, the binding of HAT1 (Fig. 6C) and CBP (Fig. 6D) on the IL-6 promoter was also inhibited in response to anacardiac acid treatment. However, no difference in HDAC1 binding on IL-6 promoter was observed between with and without anacardiac acid treatment in normal and OA SF (Fig. S5). The results from quantitative RT-PCR and western blot analysis revealed that both the mRNA (Fig. 6E) and protein (Fig. 6F) levels of IL-6 in normal and OA SF could be significantly suppressed after decreasing the histone acetylation of the IL-6 promoter. The supernatants of normal and OA SF with and without anacardic acid treatment were collected and ELISA was employed to measure IL-6 concentration. The results revealed the significantly lower levels of IL-6 secretion from normal and OA SF with anacardic acid treatment, compared with vehicle treatment (Fig. 6G).

## Discussion

The chronic inflammation of the synovial membrane plays a central role in the OA pathophysiology via a complex cytokine network. These factors initiate the destruction and loss of articular tissues. IL-6 is one of the most important players in inflammation. Previous studies have suggested that IL-6 activates osteoclasts and stimulates the synovium to produce MMPs responsible for cartilage destruction in OA^8^. A clinical trial indicated that IL-6 is involved in the pathophysiology of OA^17^,^18^. OA patients have an elevated level of IL-6 in whole blood compared with normal subjects^19^. Also, it should be noted that chondrocytes produce low level of IL-6 under normal conditions. In addition, the results from the present study revealed IL-6 over-expression in synovial fibroblasts and synovial fluid from OA patients. Therefore, these data suggest that IL-6 is involved in OA pathogenesis.

To elucidate the molecular mechanisms underlying IL-6 over-production in OA, several studies have investigated the factors upstream of IL-6, including adiponectin^20^, CCN4^21^, SDF-1/CXCR4^22^, leptin^23^, cyclin-dependent kinase inhibitor^24^ and CTGF^25^. Moreover, several consensus sequences for the binding of NF-κB, CREB, NF-IL-6, and AP-1 in the promoter of the IL-6, have been identified, which are able to induce IL-6 expression in response to various exogeneous stimuli^26^,^27^. By blocking signaling pathways upstream of IL-6 in OA, it is possible to suppress IL-6 over-expression. However, it might be a risk to block some signaling pathways upstream of IL-6 in order to suppress IL-6 over-production, as those signaling pathways are possible to have biological functions in some important biological activities. In this sense, it seems a better way to suppress the expression of IL-6 *per se*, which requires our deep understanding of the mechanisms underlying IL-6 expression. Mammalian gene expression requires the loosening of the chromatin structure, exposure of the promoter region and the binding of transcriptional factors and RNA polymerase, which is regulated by epigenetic modifications. Histone modifications, including acetylation, methylation and adenylation, ubiquitinoylation, ADP ribosylation and phosphorylation, determine the loosening of chromatin, and DNA methylation determines the accessibility of the promoter region. Thus, investigating the mechanisms underlying how epigenetic modifications regulate gene expression can provide deeper insight into gene expression regulation.

In the present study, DNA hypo-methylation and histone hyper-acetylation in the IL-6 promoter were observed in OA SF compared with normal SF. In addition, weaker binding of MeCP2, Dnmt3a and HDAC1 was observed in the IL-6 promoter in OA SF compared with normal SF. In mammals, DNA methylation primarily occurs at CpG dinucleotides, which are often observed in clusters called CpG islands generally located in the gene promoter. The methylation of CpG islands leads to stable and heritable transcriptional silencing through the binding of methyl-DNA-specific proteins to methylated CpG islands. Further, methyl-DNA-specific proteins attract histone-modifying enzymes, which establish a silenced chromatin state. Generally, lysine acetylation by histone acetyltransferases marks transcriptionally active regions. In contrast, histone deacetylases (HDACs) catalyze lysine deacetylation, and the hypoacetylated histones are primarily associated with transcriptionally inactive regions. We noticed that MeCP2 binding was reduced in regions 1 and 2 of the IL-6 promoter yet it was not for region 3 whereas hypomethylation was present in all three regions (Fig. 2D). This result indicated that MeCP2 binding was not only dependent on CpG methylation within DNA sequence and the function of MeCP2 was not only methylated CpG binding. More importantly, MeCP2 provided a binding platform to attract histone-modifying enzymes. It makes sense to look DNA methylation, histone modifications, MeCP2 binding and histone-modifying enzymes binding as a whole which are cooperated to control chromatin structure and gene transcription. This point was supported by the results of current study that just as MeCP2 binding pattern in region 3, there was also no difference in the binding of HDAC1, HAT1 and CBP in region 3 of IL-6 promoter between normal and OA samples (Fig. 3C,D and E).

Dnmt family contains Dnmt1, Dnmt3a, and Dnmt3b. Dnmt1 is a maintenance enzyme that binds hemi-methylated DNA. Dnmt3a and Dnmt3b work as *de novo* Dnmt^28^. Unlike Dnmt1 and Dnmt3a, Dnmt3b expression is relatively low in cartilage^29^. Although generally it is not easy to observe the significant changes in global DNA methylation levels or Dnmt1 and Dnmt3a levels in OA chondrocytes^29^, hypomethylation of specific CpG sites and resulted increased expression of coded genes has been reported within the gene promoters including metalloproteinase (MMP)-13 and ADAMTS-4 in end-stage OA chondrocytes^11^,^30^,^31^,^32^. Recently, several molecules are reported to change the methylation status of specific genes in chondrocytes. Glucosamine and BAY 11-7082, an NF-kB inhibitor, inhibit cytokine-induced demethylation of a specific CpG site in the IL-1β promoter, which was associated with downregulation of IL-1β in human articular chondrocytes^33^. Inflammatory cytokines can modify the DNA methylation status at key CpG sites, resulting in long-term induction of IL-1β in human articular chondrocytes^34^.

The effect of histone acetylations on chondrocyte phenotype has been studied at the level of HDACs. Specific HDACs has been reported to be involved in different processes and to target different chondrocyte-specific genes^35^,^36^. HDAC 1 and 2 expression are enhanced in OA cartilage, compared to normal samples^36^. In addition, HDAC1- and HDAC2-mediated gene repression of target gene such as COL2A1 involves their recruitment by Snail transcription factor^36^. A correlation between MMP13 and HDAC7 gene expression has been reported in human knee osteoarthritis^37^. HDAC4 prevents premature chondrocyte hypertrophy by interacting with Runx2 and blocking its activity^38^. Although HDAC inhibitors were reported to inhibit the expression of specific MMPs, HDAC inhibitors as a potential treatment for OA has been questioned by other findings showing that HDAC inhibitors also downregulate COL2A1 expression^39^.

Epigenetic modifications are intensely involved in IL-6 expression in many human diseases. For example, in a CCl4-injured liver rat model, the administration of chorionic plate-derived mesenchymal stem cells promotes IL-6/STAT3 signaling through a decrease in the methylation of IL-6/STAT3 promoters, thereby inducing the proliferation of hepatic cells^40^. Epigenetic modification by Smyd2-mediated H3K36 dimethylation at IL-6 promoters plays an important role in the regulation of macrophage activation during inflammation^41^. Rosenzweig JM *et al*. showed a novel role for the zinc finger transcription factor Kruppel-like factor 4 (KLF4) in the modulation of the inflammatory immune response by the regulation of IL-6^42^. The data indicated that KLF4 has a dual function. First, KLF4 acts as a transcription factor binding to and activating the IL-6 promoter at specific binding sites. KLF4 also plays a role in the chromatin remodeling of the IL-6 promoter, as cells deficient in KLF4 exhibited relative hypoacetylation^42^.

However, in OA, a rare report addresses the role of epigenetic modifications in IL-6 over-expression. In a similar study, the authors reported the role of histone modifications in elevated IL-6 production in rheumatoid arthritis synovial fibroblasts (RASFs). The level of histone H3 acetylation (H3ac) in the IL-6 promoter was significantly higher in RASFs than in osteoarthritis (OA) SFs^43^. Furthermore, curcumin, a histone acetyltransferase (HAT) inhibitor, significantly reduced the level of H3ac in the IL-6 promoter and decreased IL-6 mRNA expression and IL-6 protein secretion in RASFs^43^. To our knowledge, the present study is the first to evaluate the role of epigenetic modifications in the regulation of IL-6 over-expression in OA.

More importantly, in the present study, we showed not only which epigenetic modifications (DNA hypo-methylation and histone acetylation) were involved in IL-6 overexpression in OA SF but also which of these epigenetic modifications were not involved in IL-6 regulation in OA SF. No differences in the status of H3K9 di-methylation and H3K27 tri-methylation in the IL-6 promoter region were observed between normal and OA SF. Both H3K9 and K27 methylation are inactive chromatin marks. K9 methylation recruits heterochromatin-associated protein-1 to establish heterochromatic regions^44^, whereas K27 tri-methylation is involved in PcG-mediated gene silencing^45^. The data of present study suggest that compared with DNA methylation and histone acetylation, H3K9 di-methylation and H3K27 tri-methylation may not be the main target to regulate IL-6 expression.

In an interesting study conducted by Noss EH *et al*., the results showed that human RA and OA synovial fibroblasts derived from independent donors reproducibly segregated into low, medium, and high IL-6 producers, independent of stimulus, cell passage, or disease state. Further, high-fibroblast IL-6 expression was significantly associated with the IL-6 proximal promoter single nucleotide polymorphism (SNP) rs1800795 minor allele (CC) genotype. In the current study, IL-6 expression was also elevated in OA SF compared with normal samples. Since the main interest of the current study is epigenetic modifications of IL-6 in OA, the SNP described above was not genotyped within IL-6 proximal promoter. Currently the exact occurrence of this SNP in human has not been reported. Therefore, it is difficult to assess the contribution of SNP to IL-6 upregulation in OA samples in the current study. However, at least, our results suggested that DNA hypomethylation and histone hyperacetylation play a role in IL-6 upregulation in OA SF. In fact, genetic and epigenetic factors can interact mutually to regulate gene expression and disease susceptibility. Epigenetic modifications determine the penetrance of genetic susceptibility by modulating the expression of a gene loading a susceptibility allele^46^,^47^. For example, the rs143383 C/T SNP in the 5’-UTR of growth/differentiation factor 5 (GDF5) is involved in OA susceptibility. DNA methylation disrupts the expression balance between the C and T alleles and thereby increases the susceptibility to OA. On the other hand, epigenetic changes may contribute to disease risk through regulating the effect of genetic variation on disease severity as observed in heavy metals disrupting DNA methylation and chromatin structure, and anti-androgenic toxins altering DNA methylation and thereby decreasing male fertility^48^,^49^.

In the present study, we attempted to suppress IL-6 overexpression in OA SF with alterations in epigenetic modifications using two methods: gene engineering and treatment with exogenous inhibitors. Dnmt3a-overexpression caused DNA hyper-methylation and anacardic acid treatment caused H3 and H4 tail hypo-acetylation. In response to these treatments, IL-6 overexpression in OA SF was suppressed. Some anti-methylation agents have been used to tumors treatments^50^, and perhaps these agents can be also used in OA; however, the effects of these compounds are widespread, which limits their potential utility in clinical conditions. Agents that specifically modulate the epigenetic modifications of certain genes would be of much greater use. In the present study, we cannot exclude the widespread effects of Dnmt3a overexpression and anacardic acid treatment. However, we confirmed that IL-6 promoter regions have remarkable sensitivity to epigenetic treatments and that the overall effect on OA SF was the suppression of IL-6 over-expression.

In summary, IL-6 was over-expressed in SF from OA patients compared with normal donors. DNA hypomethylation and histone hyperacetylation were observed in the IL-6 promoter region in OA SF compared with normal SF. No differences in the status of H3K9 di-methylation, H3K27 tri-methylation and H3K4 tri-methylation in the IL-6 promoter region were observed between normal and OA SF. Through Dnmt overexpression and anacardic acid treatment to increase DNA methylation and decrease histone acetylation, IL-6 over-expression in OA SF was suppressed. These observations provide deeper insight into the pathophysiology of OA and can be used to design new drugs and develop new therapeutic methods to treat OA.

## Materials and Methods

### Cell culture

Written informed consent was obtained from all patients who were recruited between January and September 2015, and the study was approved by Ethics Review Committee of Shanghai Ninth People’s Hospital. The methods were carried out in accordance with the relevant guidelines and regulations of Ethics Review Committee of Shanghai Ninth People’s Hospital. Human SF was isolated through the collagenase treatment of synovial tissue from 20 OA patients receiving knee-replacement surgery and 15 samples of non-arthritic synovial tissues obtained during arthroscopy. Briefly, fresh synovial tissues were minced and digested by collagenase and DNase solution. Isolated cells were filtered through 70-μm nylon filters. The cells were plated on cell culture dishes in 5% CO_2_ in DMEM (Life Technologies, Grand Island, NY, USA) supplemented with 10% fetal bovine serum (FBS), 100 U/ml penicillin, and 100 μg/ml streptomycin. Fibroblasts between passage 4 to 9 were used for the experiments.

### Real-time PCR

Total RNA was harvested from SF using the RNeasy Mini Kit (Qiagen, Valencia, CA, USA). For RT-PCR, cDNA was reverse-transcribed from 1 μg of total RNA. Quantitative PCR was performed using SYBR Green PCR Master Mix (Takara Bio Inc., Otsu, Japan) on ABI Prism 7500 PCR machine (Applied BioSystems, Foster City, CA, USA). The following cycling conditions were used: 94 °C for 30 s, followed by 40 cycles of 94 °C for 5 s and 60 °C for 34 s. The semi-quantitative 2^−ΔΔCt^ method was employed to calculate the relative expression level of target gene. β-actin was used as loading control. Primer sequences: IL-6: 5′-CAATATTAGAGTCTCAACCCCCA-3′ (forward), 5′-CCGTCGAGGATGTACCGAAT-3′ (reverse); Dnmt1: 5′-CCTAGCCCCAGGATTACAAGG-3′ (forward), 5′-ACTCATCCGATTTGGCTCTTTC-3′ (reverse); Dnmt3a: 5′-CACCGGCCATACGGTGG-3′ (forward), 5′-CAGCAGCCATTTTCCACTGC-3′ (reverse); HDAC1: 5′-CGCCCTCACAAAGCCAATG-3′ (forward), 5′-CTGCTTGCTGTACTCCGACA-3′ (reverse); HAT1: 5′-TACAGCGGAAGATCCATCCAA-3′ (forward), 5′-CTGTTGTGCCTCTATCGCCA-3′ (reverse); GAPDH: 5′-GGAGCGAGATCCCTCCAAAAT-3′ (forward), 5′-GGCTGTTGTCATACTTCTCATGG-3′ (reverse).

### Western blot

Cells were lysed on ice for 30 min in a buffer containing 50 mM Tris-HCl, pH 7.4, 150 mM NaCl, 1% Nonidet P-40, and 0.1% SDS supplemented with protease inhibitors (10 g/ml leupeptin, 10 g/ml pepstatin A, and 10 g/ml aprotinin). The proteins were separated by SDS-PAGE, transferred to a nitrocellulose membrane, and detected using anti-IL-6 (#12912, Cell Signaling Technology, Danvers, MA, USA), anti-HA tag (#3724, CST), and anti-β-actin (#3700, CST) antibodies. The proteins were visualized using an enhanced chemiluminescence system (GE Healthcare, Piscataway, NJ, USA).

### ELISA

Normal and OA SFs were cultured in complete medium for 24 h, then the complete medium was replaced by serum-free medium. After 2 h, the conditioned medium was harvested, centrifuged to remove particulate matter, and stored at −20 °C until analyzed. Quantitation of IL-6 secreted into the medium was determined using a quantitative sandwich ELISA for human IL-6 according to the manufacturer’s instructions (R&D Systems, Minneapolis, MN, USA).

### DNA isolation and bisulfite sequencing PCR

Five microgram genomic DNA of human SF was denatured by 2 M NaOH for 15 min at 50 °C. 2% low-melting agarose were subsequently added to the DNA solution, and agarose beads were formed after pipetting 15-μl DNA/agarose mixture into cold mineral oil. The DNA/agarose beads were treated by freshly prepared hydroxyquinone (10 mM; Sigma) and sodium bisulfite (40.5%, pH 5; Sigma) at 50 °C for 16 h under mineral oil. The reaction was stopped by 0.3 M NaOH for 10 min at room temperature.

The PCR amplifications were performed in 25 μl reactions containing one agarose/DNA bead. The following primer sequences were used: Region 1: 5′-TTTTTATATTAAAGAATTTT-3′ (forward), 5′-TTTATTTATTAAATATGGTGT-3′ (reverse); Region 2: 5′-TAGGTGAAGAAAGTGGTAGA-3′ (forward), 5′-GATTAGATTAATAGGTTAGAA-3′ (reverse) and Region 3: 5′-TTTTTAGTTTTGGAATTGTT-3′ (forward), 5′-AGGTAATATTAGGAGTAGTTTT-3′ (reverse). The PCR products were separated by agarose gel, purified and cloned into the pMD18-T vector system (Takara). Fifteen clones of each sample were sequenced, and the sense strands were used to evaluate CpG site methylation status.

### Chromatin immunoprecipitation (ChIP)

The cells were cross-linked with 1% formaldehyde for 10 min at 37 °C, and crude nuclei were recovered. The crude nuclei were sonicated to produce 500 bp chromatin fragments. The following antibodies were used in the ChIP assay: MeCP2, H3K9 di-methylation, HDAC1, Dnmt1, Dnmt3a, SETDB1, MLL2 (Abcam), H3K9/K14 acetylation, H4K12 acetylation, H3K4 tri-methylation, H3K27 tri-methylation (Upstate), HAT1 (GeneTex), CBP (Abcam), p300 (Abcam) and Ezh2 (Cell Signaling Technology, Danvers, MA, USA). IgG (Sigma) was used as a negative control. For each ChIP assay, 5 μg antibodies were added, and the samples were incubated overnight at 4 °C. The ChIP and input DNA samples were quantified using quantitative PCR. Primer sequences used in ChIP-qPCR: Region1: 5′-TTTTCACACCAAAGAATCCC-3′ (forward), 5′-CTTATTTACCAAACATGGTGT-3′ (reverse); Region2: 5′-CAGGTGAAGAAAGTGGCAGA-3′ (forward), 5′-AAGATCGGACAATTAGACCAG-3′ (reverse); Region3: 5′-TCCTTAGCCCTGGAACTGCC-3′ (forward), 5′-AGGCAACACCAGGAGCAGCCCC-3′ (reverse).

### *In vitro* methylation and luciferase reporter assay

M.SssI (New England Biolabs, Ipswich, MA, USA) was employed to *in vitro* methylate CpG sites for 6 h at 37 °C. The treated DNA fragments and vectors were ligated and subsequently purified using phenol/chloroform extraction and ethanol precipitation.

Cells were plated onto 24-well plates. Transient transfections were performed using Fugene6 (Roche); the phRL-SV40 vector (Promega, Madison, WI, USA) was used as control for transfection efficiency. Forty-eight hours after transfection, both firefly and Renilla luciferase activities were measured using a dual-luciferase reporter assay system (Promega). The relative luciferase units (RLUs), the ratios of firefly to Renilla luciferase activities, were subsequently calculated.

### Lentivirus

Lentiviral vectors containing the coding sequences of Dnmt3a were purchased from Genecopoeia (Rockville, MD, USA). 293T cells were plated onto a 10-cm dish, and the transfection mixture was added directly to the culture medium at 70–80% confluence. Following transfection, the samples were incubated in a CO_2_ incubator at 37 °C for 48 h and the culture medium containing virus particle was subsequently collected.

### Statistical analysis

For the data with abnormal distribution, the nonparametric method was employed. For the data with normal distribution, Student’s t-test was used for two-sample comparisons and one-way ANOVA was used for multiple comparisons. Tukey’s test was employed to evaluate significant differences using ANOVA. P values < 0.05 were regarded as significant. All data are presented as the means ± SD unless otherwise specified.

## Additional Information

**How to cite this article:** Yang, F. *et al*. Epigenetic modifications of interleukin-6 in synovial fibroblasts from osteoarthritis patients. *Sci. Rep.* **7**, 43592; doi: 10.1038/srep43592 (2017).

**Publisher's note:** Springer Nature remains neutral with regard to jurisdictional claims in published maps and institutional affiliations.

## Supplementary Material

Supplementary Data
