# Supplementary material for: Epigenetic modifications of interleukin-6 in synovial fibroblasts from osteoarthritis patients
Source: Sci Rep. 2017 Mar 6;7:43592. doi: 10.1038/srep43592 (PMC5337936; doi:10.1038/srep43592)
Supplement: Supplementary Data [file srep43592-s1.pdf]

Epigenetic modifications of interleukin-6 in synovial fibroblasts from osteoarthritis patients

Fei Yang[1], Song Zhou[2], Chuandong Wang[3], Yan Huang[3], Huiwu Li[1], You Wang[1], Zhenan Zhu[1], Jian Tang[1]*, Mengning Yan[1]*

1 Shanghai Key Laboratory of Orthopaedic Implants, Department of Orthopaedics, Shanghai Ninth People's Hospital, Shanghai Jiao Tong University School of Medicine, 639 Zhizaoju Road, Shanghai 200011, PR China

2 Department of Orthopaedics, Linyi People's Hospital, 27 East Jiefang Road , Linyi 276000, PR China

3 The Key Laboratory of Stem Cell Biology, Institute of Health Sciences, Shanghai Institutes for Biological Sciences, Chinese Academy of Sciences & Shanghai Jiao Tong University School of Medicine, 320 Yueyang Road, Shanghai 200031, PR China

Running title: IL-6 epigenetic modifications in OA

* Corresponding authors:

Mengning Yan

Tel/Fax: +86-21-63139920；E-mail address: y_mengning@sina.com

Jian Tang

Tel/Fax: +86-21-63139920；E-mail address: shjytangjian@sohu.com

GAAACCATCCAGCCATCCTCCCCCATTTTCATTTTCACACCAAAGAATCCC
ACCGCGGCAGAGGACCACCGTCTCTGTTTAGACAATCGGTGAAGAATGGA
TGACCTCACTTTCCCCAACAGGCGGGTCCTGAAATGTTATGCACGAAACA
AAACTTGAGTAAATGCCCAACAGAGGTCACTGTTTTATCGATCTTGAAGA
GATCTCTTCTTAGCAAAGCAAAGAAACCGATTGTGAAGGTAACACCATGT
TTGGTAAATAAGTGTTTTGGTGTTGTGCAAGGGTCTGGTTTCAGCCTGAA
GCCATCTCAGAGCTGTCTGGGTCTCTGGAGACTGGAGGGACAACCTAGTC
TAGAGCCCATTTGCATGAGACCAAGGATCCTCCTGCAAGAGACACCATCC
TGAGGGAAGAGGGCTTCTGAACCAGCTTGACCCAATAAGAAATTCTTGGG
TGCCGACGCGGAAGCAGATTCAGAGCCTAGAGCCGTGCCTGCGTCCGTAG
TTTCCTTCTAGCTTCTTTTGATTTCAAATCAAGACTTACAGGGAGAGGGAG
CGATAAACACAAACTCTGCAAGATGCCACAAGGTCCTCCTTTGACATCCC
CAACAAAGAGGTGAGTAGTATTCTCCCCCTTTCTGCCCTGAACCAAGTGG
GCTTCAGTAATTTCAGGGCTCCAGGAGACCTGGGGCCCATGCAGGTGCCC
CAGTGAAACAGTGGTGAAGAGACTCAGTGGCAATGGGGAGAGCACTGGC
AGCACAAGGCAAACCTCTGGCACAGAGAGCAAAGTCCTCACTGGGAGGA
TTCCCAAGGGGTCACTTGGGAGAGGGCAGGGCAGCAGCCAACCTCCTCT
AAGTGGGCTGAAGCAGGTGAAGAAAGTGGCAGAAGCCACGCGGTGGCA
AAAAGGAGTCACACACTCCACCTGGAGACGCCTTGAAGTAACTGCACGA
AATTTGAGGATGGCCAGGCAGTTCTACAACAGCCGCTCACAGGGAGAGC
CAGAACACAGAAGAACTCAGATGACTGGTAGTATTACCTTCTTCATAATCC
CAGGCTTGGGGGGCTGCGATGGAGTCAGAGGAAACTCAGTTCAGAACAT
CTTTGGTTTTTACAAATACAAATTAACTGGAACGCTAAATTCTAGCCTGTTA
ATCTGGTCACTGAAAAAAAATTTTTTTTTTCAAAAAACATAGCTTTAGC
TTATTTTTTTCTCTTTGTAAAACTTCGTGCATGACTTCAGCTTTACTCTTT
GTCAAGACATGCCAAAGTGCTGAGTCACTAATAAAAGAAAAAAAGAAAG
TAAAGGAAGAGTGGTTCTGCTTCTTAGCGCTAGCCTCAATGACGACCTAA
GCTGCACTTTTCCCCCTAGTTGTGTCTTGCCATGCTAAAGGACGTCACATT
GCACAATCTTAATAAGGTTTCCAATCAGCCCCACCCGCTCTGGCCCCACCC
TCACCCTCCAACAAAGATTTATCAAATGTGGGATTTTCCCATGAGTCTCAA

TATTAGAGTCTCAACCCCCAATAAATATAGGACTGGAGATGTCTGAGGCTC
ATTCTGCCCTCGAGCCCACCGGGAACGAAAGAGAAGCTCTATCTCCCCTC
CAGGAGCCCAGCTATGAACTCCTTCTCCACAAGTAAGTGCAGGAAATCCT
TAGCCCTGGAACTGCCAGCGGCGGTCGAGCCCTGTGTGAGGGAGGGGTG
TGTGGCCCAGGGAGGGCTGGCGGGCGGCCAGCAGCAGAGGCAGGCTCCC
AGCTGTGCTGTCAGCTCACCCCTGCGCTCGCTCCCCTCCGGCACAGGCGC
CTTCGGTCCAGTTGCCTTCTCCCTGGGGCTGCTCCTGGTGTTGCCTGCTGC
CTTCCCTGCCCCAGTACCCCCAGGAGAAGATTCCAAAGATGTAGCCGCCC
CACACAGACAGCCACTCACCTCTTCAGAACGAATTGACAAACAAATTCGG
TACATCCTCGACGGCATCTCAGCCCTGAGAAAGGAGGTGGGTAGGCTT

Transcription start site **CpG site** **Forward primer** **Reverse primer**

Figure S1

Figure S1. Human IL-6 promoter sequence investigated in the current study. The DNA sequence of -1500bp~+500bp around human IL-6 transcription start site was shown. Transcription start site was labeled in red, CpG site was labeled in yellow, forward primer site was labeled in gray and reverse primer site was labeled in green.

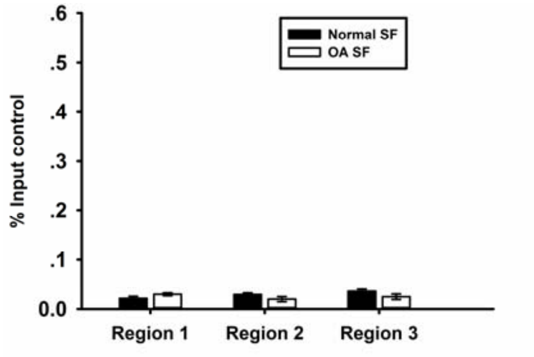

Figure S2

Figure S2. Negative control for ChIP assay. ChIP assays using IgG antibody in the region 1, 2, and 3 of human IL-6 were performed in normal and OA synovial fibroblasts (SF). The results were expressed as the percentage in input control. Data were shown as mean±SD.

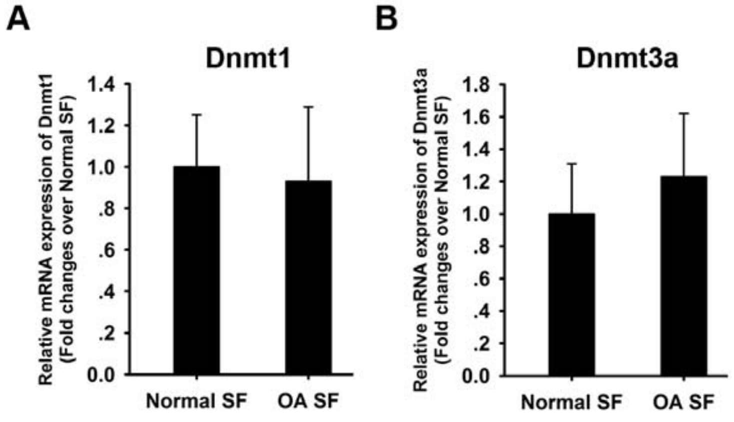

Figure S3

Figure S3. Dnmt1 and Dnmt3a mRNA expression in synovial fibroblasts from OA patients. Synovial fibroblasts (SF) were isolated from non-arthritic donors and OA patients and cultured *in vitro*. Total RNA was isolated for quantitative RT-PCR using Dnmt1 **(A)** and Dnmt3a **(B)** primers. β-actin was used as an internal control. The results are expressed as the fold-change relative to normal SF. Data are shown as the means±SD.

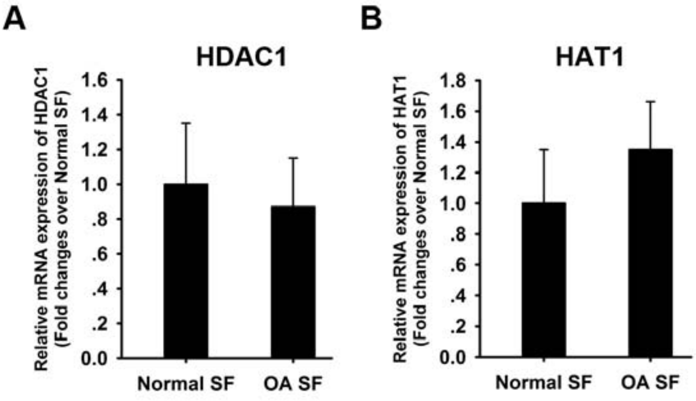

Figure S4

Figure S4. HDAC1 and HAT1 mRNA expression in synovial fibroblasts from OA patients. Synovial fibroblasts (SF) were isolated from non-arthritic donors and OA patients and cultured *in vitro*. Total RNA was isolated for quantitative RT-PCR using HDAC1 **(A)** and HAT1 **(B)** primers. β-actin was used as an internal control. The results are expressed as the fold-change relative to normal SF. Data are shown as the means±SD.

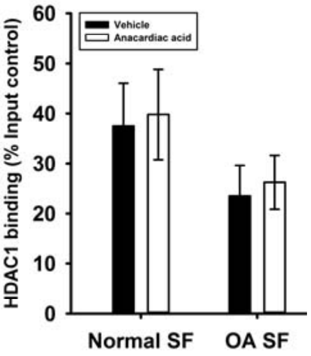

Figure S5

Figure S5. HDAC1 binding in region 2 of the IL-6 promoter in response to anacardiac acid treatment. Normal and OA synovial fibroblasts (SF) were treated with anacardic acid (4 μM). ChIP was performed to measure HDAC1 occupancy in region 2 of the IL-6 promoter. The results were normalized to the percentage of the input control. Data were shown as mean±SD.
